# The laparoscopic modified Sugarbaker technique is safe and has a low recurrence rate: a multicenter cohort study

**DOI:** 10.1007/s00464-012-2464-4

**Published:** 2012-10-10

**Authors:** B. M. E. Hansson, S. Morales-Conde, T. Mussack, J. Valdes, F. E. Muysoms, R. P. Bleichrodt

**Affiliations:** 1 Department of Surgery, Canisius-Wilhelmina Hospital, P.O. Box 9015, 6500 GS Nijmegen, The Netherlands; 2 Unit of Innovation in Minimal Invasive Surgery, University Hospital Virgen del Rocio, Seville, Spain; 3 Department of Surgery, Klinikum der Universität München, Campus Innenstadt, Munich, Germany; 4 Department of Colorectal Surgery, University Hospital Virgen Macarena, Seville, Spain; 5 Department of General Surgery, Maria Middelares, Gent, Belgium

**Keywords:** Parastomal, Hernia, Sugarbaker, ePTFE, GoreTex, Mesh repair

## Abstract

**Background:**

Parastomal hernia is a frequent complication of intestinal stomata. Mesh repair gives the best results, with the mesh inserted via laparotomy or laparoscopically. It was the aim of this retrospective multicenter study to determine the early and late results of the laparoscopically performed, modified Sugarbaker technique with ePTFE mesh.

**Methods:**

From 2005 to 2010, a total of 61 consecutive patients (mean age = 61 years), with a symptomatic parastomal hernia, underwent laparoscopic repair using the modified Sugarbaker technique with ePTFE mesh. Fifty-five patients had a colostomy, 4 patients an ileostomy, and 2 a urostomy according to Bricker. The records of the patients were reviewed with respect to patient characteristics, postoperative morbidity, and mortality. All patients underwent physical examination after a follow-up of at least 1 year to detect a recurrent hernia. Morbidity rate was 19 % and included wound infection (*n* = 1), ileus (*n* = 2), trocar site bleeding (*n* = 2), reintervention (*n* = 2), and pneumonia (*n* = 1). One patient died in the postoperative period due to metastasis of lung carcinoma that caused bowel obstruction. Concomitant incisional hernias were detected in 25 of 61 patients (41 %) and could be repaired at the same time in all cases. A recurrent hernia was found in three patients at physical examination, and in one patient an asymptomatic recurrence was found on a CT scan. The overall recurrence rate was 6.6 % after a mean follow-up of 26 months.

**Conclusion:**

The laparoscopic Sugarbaker technique is a safe procedure for repairing parastomal hernias. In our study, the overall morbidity was 19 % and the recurrence rate was 6.6 % after a mean follow-up of 26 months. Moreover, the laparoscopic approach revealed concomitant hernias in 41 % of the patients, which could be repaired successfully at the same time.

A parastomal hernia is an incisional hernia related to the presence of an enterostomy [1]. It is a common complication of stoma formation and the reported incidence varies from 3 to 39 % for colostomies and from 0 to 6 % for ileostomies [2]. Most parastomal hernias are asymptomatic and therefore can be treated conservatively. Indications for surgery are ill-fitting appliances causing leakage, pain, discomfort, and cosmetic complaints [3]. Urgent treatment is indicated when incarceration or strangulation of hernia content occurs.

Surgical treatment options are relocation of the stoma, or repair with or without the use of prosthetic material via an open or a laparoscopic approach. Recently, a systematic review of surgical repair of parastomal hernias was published by Hansson et al. [4]. It was concluded that suture repair should be regarded as outdated because of the high recurrence rate of 69.4 %. Synthetic mesh repair had significantly better results with respect to wound infection and recurrence rate. Depending on technique and placement, recurrence rates after mesh repair varied between 6.9 and 17.8 %. The overall mesh infection rate was 2.4 %. The recurrence rate was similar in patients in whom the mesh was implanted on the fascia (onlay), preperitoneally behind the rectus muscle, or intraperitoneally, although the onlay position tended to have a higher recurrence rate.

The preperitoneal, retromuscular, or intraperitoneal positions of meshes are biomechanically more attractive and therefore favored by most surgeons. In the review of Hansson et al. [4], it was found that the modified Sugarbaker technique had the best results with respect to recurrence rate. In 1985, Sugarbaker described his technique for parastomal hernia repair [5]. Via a laparotomy, the trephine opening is covered with an intraperitoneally placed prosthetic mesh that is sutured to the fascial edge. The bowel is lateralized, passing from the hernia sac between the abdominal wall and the prosthesis into the peritoneal cavity. As we have learned from incisional hernia repair, an overlap of 3–5 cm between the mesh and the adjacent fascia is mandatory to prevent recurrent hernias [6]. Therefore, the Sugarbaker technique was modified around the trephine opening to guarantee an adequate overlap between the mesh and the fascia (Figs. 1, 2).

**Fig. 1:**
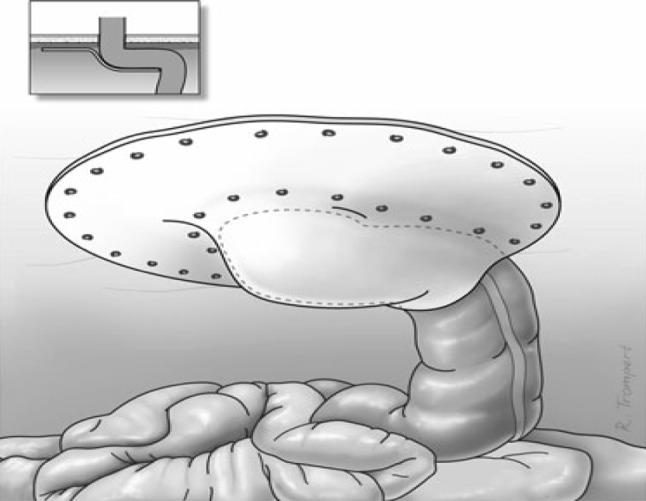
Laparoscopic Sugarbaker technique

**Fig. 2:**
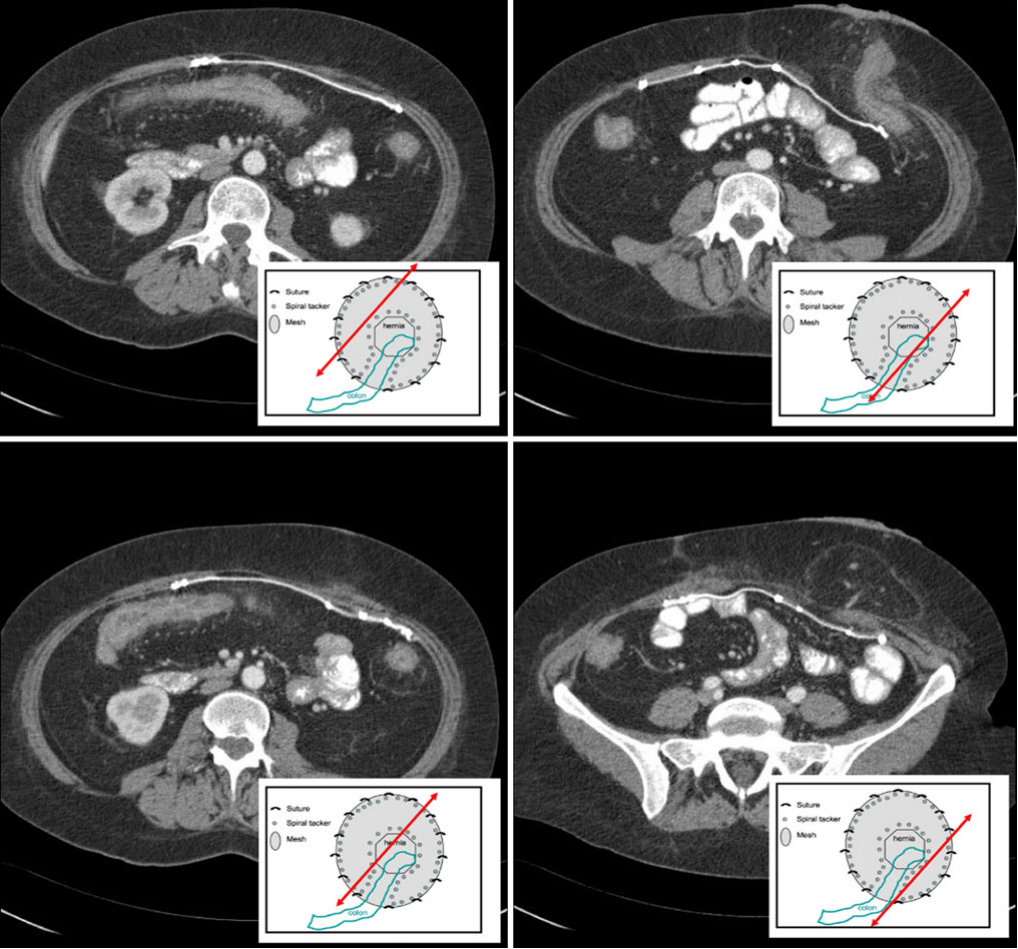
Postoperative multislice CT scan after laparoscopic Sugarbaker repair

Laparoscopic repair of an incisional hernia is favored by many surgeons because of a low infection rate of 0.7 % [7]. Meta-analysis of all randomized controlled trials performed by Forbes et al. [8] showed significantly lower wound and mesh infection rates in the laparoscopic group. Another potential advantage of the laparoscopic approach is that concomitant incisional hernias can be detected and repaired at the same time. In a recent meta-analysis [4], the recurrence rate of the laparoscopic Sugarbaker repair was found to be 11.6 % (95 % CI = 6.4–18.0) in a group of 110 patients from six studies. Berger et al. [9] reported on the use of a sandwich technique that combines the Sugarbaker and keyhole techniques. After a median follow-up of 20 (range = 6–48) months, one of 47 (2.1 %) patients had a recurrent hernia. Recently, Mizrahi et al. [10] published data on the keyhole technique similar to that of Hansson et al. [4] published previously. Recurrences up to 46.4 % were reported by Mizrahi et al.

The aim of the present study was to determine the results of the laparoscopically performed Sugarbaker technique for the repair of parastomal hernias done at four European centers with extensive experience in laparoscopy and laparoscopic hernia repair.

## Patients and methods

A retrospective multicenter study was performed to determine the results of laparoscopic repair of parastomal hernias via a modified Sugarbaker technique. All consecutive patients who were operated on in the four participating centers between May 2005 and June 2010 were included in the study. The following data were extracted from the records: age, BMI, size of defect, comorbidities, ASA score, indication for surgery, technical details of the operation [i.e., adhesion score; size of the trephine opening, calculated as the area of an ellipse using the formula π × (0.5 × length) × (0.5 × width), intraoperative complications, and operating time], postoperative mortality and morbidity, duration of follow-up, and the presence of a recurrent hernia. Adhesions were scored following Zühlke [11]: grade 1, filmy adhesion, easy to separate by blunt dissection; grade 2, stronger adhesion, blunt dissection possible, partly sharp dissection necessary; grade 3, strong adhesions, lysis possible by sharp dissection only; and grade 4, very strong adhesions, lysis possible by sharp dissection only, organs strongly attached with severe adhesions, damage of organs hardly preventable.

### Surgical technique

The patient is operated on while in the supine position with both arms placed along the body. The surgeon and the assistant stand at the contralateral site of the stoma. After application of pneumoperitoneum, one (or two) 10-mm trocar and two (or one) 5-mm trocars are introduced, as described by Muysoms [12]. A careful adhesiolysis is performed.

After freeing the adhesions, the stoma loop is completely dissected free from the fascia and the peritoneum around the trephine opening is freed from adhesions to allow an overlap of at least 4 cm between the abdominal wall and the prosthesis, around the hernia defect.

The trephine opening is covered with an intraperitoneally placed ePTFE patch (Gore-Tex Dual Mesh Biomaterial^®^, WL Gore Associates, Newark, DE, USA). The bowel is lateralized, passing from the hernia sac between the abdominal wall and the prosthesis into the peritoneal cavity. In this way a tunnel is created between the abdominal wall and the prosthesis (Fig. 1). It is of utmost importance to prevent narrowing of the bowel in the tunnel and angulation of the bowel when entering the abdominal cavity and the hernia sac. The prosthesis is fixed to the abdominal wall using the double-crown technique, as described by Morales–Conde [13]. After removal of the trocars, the 10-mm trocar opening is closed in layers.

### Follow-up

All patients were seen in the outpatient department and underwent physical examination. A recurrent hernia was defined as a recurrent or persistent bulge when the patient is standing during a Valsalva maneuver, or palpation of the fascial defect with the patient in the supine position [14]. When in doubt, a CT or MRI was performed. In our study 27 patients underwent a CT or a MRI.

## Results

From May 1, 2005 to June 1, 2010, a total of 61 consecutive patients (40 women; mean age = 63 years, range = 36–83 years) were treated for a symptomatic parastomal hernia. The demographic details are listed in Table 1. All but two of the procedures were performed in an elective setting. Two procedures were done as an emergency procedure for an incarcerated hernia. A concomitant incisional hernia was present in 25 (41 %) of the patients.

**Table 1:** Demographic data

Age (mean)	63 years (range = 36–83)
Gender	M: 40 (65.6 %); F: 21 (34.4 %)
BMI	30.9 (range = 18.6–51)
ASA	I, 5 (8.2 %); II, 34 (55.7 %); III, 20 (32.8 %); IV, 2 (3.3 %)
Comorbidity	Coronary disease, 9; diabetes, 1; COPD, 5; IBD, 2
Stoma type	Colostomy, 55; ileostomy, 4; urostomy, 2
Indication for stoma	Colorectal and anal malignancy, 43; bladder carcinoma, 2; IBD, 6 (CU, 4; Crohn’s disease, 2); diverticulitis, 6; incontinence, 3; benign rectal stenosis, 1
Previous PSH repair	Open mesh repair, 7; primary suture repair, 1; laparoscopic keyhole technique, 3
Symptoms	Stoma care problems, 10; intermittent bowel obstruction, 18; pain, 31; problems with bowel irrigation, 2; cosmetic complaints, 26; incarceration, 2

*PSH* parastomal hernia, *COPD* chronic obstructive pulmonary disease, *IBD* inflammatory bowel disease

Of the 61 patients, 55 had had a colostomy, 4 an ileostomy, and 2 a urostomy according to Bricker. Enterostomies were created for colorectal and anal malignancies in 43 patients, bladder cancer in 2 patients, inflammatory bowel disease in 6 patients, diverticulitis in 6 patients, incontinence in 3 patients, and benign rectal stenosis in 1 patient.

A first repair was performed in 50 patients (47 patients with a colostomy, 2 with an ileostomy, and 1 with a urostomy). Eleven patients (8 with a colostomy, 2 with an ileostomy, and 1 with a urostomy) were treated for a recurrent parastomal hernia after having an open mesh repair (*n* = 7), a laparoscopic keyhole repair (*n* = 3), or a suture repair (*n* = 1).

The indications for elective repair were stoma care problems in 10 patients, intermittent bowel obstruction in 18 patients, pain in 31 patients, problems with bowel irrigation in 2 patients, and aesthetic problems in 26 patients. The indication for emergency surgery was an incarcerated hernia with bowel obstruction in 2 patients

### Surgery

A laparoscopic Sugarbaker repair was performed in all patients. All patients had antibiotic prophylaxis with a cephalosporin. The operation was converted to an open procedure in one of the 61 patients because of an inadvertent enterotomy. The mean operating time was 111.9 min (range = 55–295 min). The mean size of the trephine opening was 31.92 cm^2^ (range = 6–169 cm^2^).

Adhesions were present in 54 of the 61 patients: grade 1 adhesions in 22 patients, grade 2 in 15, and grade 3 in 17. No severe hemorrhages were reported. A concomitant incisional hernia was found during laparoscopy in 25 patients. In all 25 cases this hernia could be repaired at the same time just by using a larger mesh or by using an additional mesh in four patients. The mean size of the mesh used was 331.54 cm^2^ (range = 225–884 cm^2^). The mesh was fixed to the abdominal wall with spiral tacks (Protack^®^, Covidien, Mansfield, MA, USA) in all 61 patients. In 27 patients cardinal sutures and in 16 patients fibrin glue was used as well, to fix the prosthesis.

One patient had a small bowel obstruction due to lung carcinoma metastasis and died 1 month after surgery. Overall morbidity was 19 % (12 patients). Surgical complications occurred in 11 patients (18 %): wound infection (*n* = 1), postoperative ileus needing insertion of a nasogastric tube (*n* = 6), and trocar site bleeding (*n* = 2). A reintervention was done in two patients. One patient had a mesh infection and the mesh was removed via a laparotomy. One patient had postoperative pneumonia. No other medical complications occurred. The mean hospital stay was 5 days (range = 1–21 days).

### Follow-up

During follow-up the mesh was removed in one patient who underwent total colectomy and ileostomy; no recurrence was detected at the time of operation.

All patients were seen in the outpatient clinic for clinical evaluation of their stoma. The mean follow-up time was 26 months. Seroma formation occurred in 12 patients (20 %) and was treated conservatively in all patients. Recurrent symptomatic hernias were found in 3 of 60 patients (5 %), including one as a result of mesh removal for infection. Recurrences occurred after 6, 10, and 20 months, respectively.

A CT or MRI scan was performed in 27 of the 60 patients after a mean follow-up of 20.4 months (range = 12–64 months). In one of the participating centers, a CT or MRI was done routinely after 1 or 2 years. Nineteen patients who had a CT or MRI had an asymptomatic hernia and the other 8 CT scans were made on indication. None of these patients had a recurrent hernia. Overall, a recurrent hernia was found in 4 of 61 patients (6.6 %).

## Discussion

The laparoscopic Sugarbaker technique is a safe procedure to repair parastomal hernias. In our study, overall morbidity was 19 % and recurrence rate was 6.6 % after a mean follow-up of 26 months.

The present study was a retrospective multicenter study; therefore, the perioperative complication rate may be underreported. The recurrence rate was determined during follow-up for stoma evaluation, at least after 1 year. All patients underwent a physical examination without imaging performed routinely. Therefore, it is reasonable to assume that the overall recurrence rate might be higher than reported.

Laparoscopic parastomal hernia repair is a safe and feasible procedure [15]. Conversion to open repair is rare. In a recent review by Hansson et al. [4], the conversion rate to open repair was 3.6 % of 363 laparoscopic repairs. Reasons for conversion were multiple dense adhesions in six patients, intraoperative full-thickness bowel injury in six patients, and an inaccessible abdomen in one patient. Iatrogenic intraoperative bowel lesions were reported in 4.1 %. Conversion rate (1.6 %) and inadvertent enterotomy rate (1.6 %) were lower is our study, probably because all procedures were done by experienced laparoscopic surgeons.

Overall morbidity in this study was 19 %, which is similar to the morbidity rate of 17.2 % (95 % CI = 13.4–21.3) for laparoscopic parastomal hernias in a recent meta-analysis [4]. Also, the complications were similar: wound infection in 3.3 % (95 % CI = 1.6–5.7), mesh infection in 2.7 % (95 % CI = 1.2–5.0), and other complications in 12.7 % (95 % CI = 9.4–16.8). Most complications resolve without further consequences; however, mesh infection often results in mesh removal and a recurrent hernia.

In our present study, all repairs were done using an ePTFE patch. At the moment, ePTFE is the most frequently used prosthetic material for parastomal hernia repair. It is soft and pliable and causes less severe adhesions to the viscera compared to polypropylene meshes [16]. If adhesions occur, the bowel can be easily dissected free from the prosthesis [17].

The hydrophobicity of ePTFE and the lack of ingrowth of fibrocollagenous tissue into the prosthesis make it vulnerable to infection [18]. Microorganisms can easily settle into the micropores of the prosthetic material, making them unreachable to granulocytes and macrophages. Therefore, infection of an ePTFE prosthesis almost always results in removal. Laparoscopic (parastomal) hernia repair is considered to be a clean operation because contact between prosthesis and bowel contents is avoided. In the only prospective series that reported on the laparoscopic repair of 55 parastomal hernias with an ePTFE patch, prosthetic infection was found in 3.6 % [19]. These results agree with those of several other studies as reviewed by Hansson et al. [4]. Although the use of an ePTFE prosthesis is safe, the authors advise against using this prosthesis in a contaminated field, e.g., after an inadvertent large bowel enterotomy.

The lack of ingrowth of fibrocollagenous tissue into this microporous structured mesh and its tendency to shrink due to intense inflammatory reaction of the host may increase the risk of reherniation [16, 20]. Initially, anchoring the patch to the adjacent fascia depends solely on sutures and tacks. Later, a fibrocollagenous envelope develops around the prosthesis, which will anchor the prosthesis to the fascia. In experimental studies it was found that the patches shrink due to retraction of the enveloping tissue. Therefore, an overlap of at least 4 cm of the fascia and the prosthesis is advocated. In clinical practice the problem may be less prominent. Schoenmaeckers et al. [21] reported that shrinkage of ePTFE in 656 patients who underwent laparoscopic hernia repair was only 7.5 % when measured by CT. This was recently confirmed by Carter et al. [22] who reported a mean shrinkage rate of 6.7 %, confirming that ePTFE has minimal contraction in the human clinical situation.

The overall recurrence rate in our series was 6.6 %. In the literature, recurrence rates for the laparoscopic Sugarbaker technique are somewhat higher. In a recent meta-analysis of six studies on 110 Sugarbaker repairs, a recurrent hernia was reported in 13 patients [11.6 % (95 % CI = 6.4–18.0)] [9, 23–27]. The recurrence rate of the keyhole technique tended to be higher than that of the Sugarbaker technique. In seven studies reporting on 160 repairs using the keyhole technique [19, 23–28], recurrences were reported in 38 patients [34.6 % (95 % CI = 15.0–27.3)]. All studies had a follow-up of at least 12 months [4]. In four series, repairs were done with either the Sugarbaker or the keyhole technique. In all studies, the recurrence rate was lower in the Sugarbaker group. Muysoms et al. [26] noted a recurrence in 2 of 13 (15 %) patients after Sugarbaker repair and in 8 of 11 (73 %) patients after keyhole repair. Craft et al. [25] reported no recurrences using the Sugarbaker technique and in one of the five repairs done with the keyhole technique. Pastor et al. [24] reported a reherniation in two of seven (28.6 %) patients after Sugarbaker repair and in two of three patients after keyhole repair.

Recurrence rates may be further reduced by using a polypropylene prosthesis that is fully incorporated into native tissue. Most surgeons are reluctant to implant polypropylene meshes into the abdomen because of its tendency to cause severe adhesions and even visceral damage, which may have serious complications and huge consequences during reoperations [29]. Berger et al. [30] used intraperitoneally placed PVDF-PP meshes (DynaMesh^®^, FEG Textiltechnik, Aachen, Germany) in 47 patients, using a combination of the Sugarbaker and keyhole techniques, better known as the sandwich technique. Only one patient developed a wound infection and three patients underwent revision: two because of stenosis and one due to an abscess. A recurrence rate of 2 % was reported.

Although a recurrence rate of 6.6 % in our study is very promising, we must keep in mind that the incidence of reherniation of incisional hernias will always increase over time, as stated by Jeekel et al. [31]. For this reason, Flum et al. [32] emphasized the importance of a follow-up of at least 5 years when comparing new techniques of hernia repair. Reviewing the literature, no report on parastomal hernia repair meets this criterion. Therefore, we intend to report long-term results on our patients after 5 and 10 years.

## Conclusion

Laparoscopic parastomal hernia repair using the Sugarbaker technique with an ePTFE mesh is safe and feasible in experienced hands. Our study shows an overall morbidity of 19 % and a recurrence rate of 6.6 % after a mean follow-up of 2 years. A laparoscopic approach revealed a concomitant incisional hernia in 41 % of the patients, which was repaired at the same time in all cases. Besides that, laparoscopy is minimally invasive to the patient’s abdominal wall, which is already at risk for herniation.
